# Pathogenic landscape of idiopathic male infertility: new insight towards its regulatory networks

**DOI:** 10.1038/npjgenmed.2016.23

**Published:** 2016-08-17

**Authors:** Narasimhan Kothandaraman, Ashok Agarwal, Muhammad Abu-Elmagd, Mohammed H Al-Qahtani

**Affiliations:** 1 American Centre for Reproductive Medicine, Glickman Urological and Kidney Institute, Cleveland Clinic, Cleveland, OH, USA; 2 Centre of Excellence in Genomic Medicine Research, King Abdulaziz University, Jeddah, Saudi Arabia; 3 Centre of Innovation in Personalized Medicine, King Abdulaziz University, Jeddah, Saudi Arabia

## Abstract

Idiopathic male infertility (IMI) affects nearly 10−15% of men in their prime reproductive age. More than 500 target genes were postulated to be associated with this disease condition through various genomic studies. The challenge is to determine the functional role of these genes and proteins that form part of a larger network leading to pathogenesis of the IMI phenotype in humans. In the current study, we have catalogued all of the genes associated with IMI from published studies, as well as looked at reactive oxygen species and antioxidant genes, the two key physiological determinants essential for normal spermatogenesis. Any imbalance in these genes through mutation, single-nucleotide polymorphisms (SNPs) or other forms could result in abnormal regulation of genes leading to infertility. SNPs catalogued in the current study, representing a third of the IMI genes, could possibly explain the various hidden factors associated with this condition. The enriched biological functions in SNPs, as well as functional analysis of IMI genes, resulted in the identification of novel gene pairs, from which we proposed new models to describe the underlying pathogenesis of this disease condition. The outcome of this study will give a new set of genes and proteins that could help explain the disease from a global perspective previously not addressed using standard approaches. Genes corresponding to proteins identified from the current study for spermatozoa and seminal plasma showed functional correlation based on their localization, which gave further confirmation of their roles in defective spermatogenesis as seen in IMI.

## Introduction

Studies from the last decade show idiopathic male infertility (IMI) as a multifactorial heterogeneous disease.^1^ Currently few modalities are available to diagnose patients with IMI and provide appropriate interventions.^2^ The effect of reactive oxygen species (ROS) and free radical-mediated damages to spermatozoa and seminal plasma is well characterized; however, their molecular ramifications on a global scale were not investigated.^3–5^ Such a study will help to access the impact of ROS on spermatogenesis in a broader perspective. Subtle changes in the proteome resulting from aberrations in the transcriptome machinery could be captured using highly sensitive mass spectrometry (MS) techniques. These changes or hidden factors will have significant impact on the final outcome of the gametogenesis.^6,7^ We hypothesized that IMI in association with ROS results from unique underlying factors at the genetic as well as proteomic level, which differs from normal parameters, laid out for diagnosing standard male infertility. These entities could manifest as (a) ROS genes, (b) single-nucleotide polymorphisms (SNPs) and (c) antioxidant (AO) genes presenting with unique signature patterns in both spermatozoid as well as seminal plasma of individuals affected with IMI. Diseased proteins coded by these candidate genes could serve as markers for understanding the molecular mechanisms as well as diagnosing and monitoring treatment options for IMI.

IMI is a much-debated terminology considering the different degrees of male infertility prevalent in the general population, with most severe forms manifested as azoospermia and asthenozoospermia.^8–10^ Current consensus on ‘idiopathic infertility’ describes the condition as ‘following an array of genetic testing to identify the disease condition the etiology remains unknown for about 40% of primary testicular failure cases’.^11^ Search for causative genetic factors has mostly focused on gene polymorphisms with limited success.^12^ Usually, infertility of unknown origin comprises both idiopathic and unexplained infertility. Men presenting with idiopathic infertility have no obvious history of fertility problems and both physical examination and endocrine laboratory testing are normal. However, semen analysis as routinely performed reveals sperm abnormalities that come alone or in combination. The reported prevalence of men with unexplained reduction of semen quality ranges from 30 to 40%. In contrast to idiopathic infertility, the term ‘unexplained infertility’ is reserved for couples in whom routine semen analysis is within normal reference values, and a definitive female infertility factor could not be identified.^13^ Though a large volume of research has been conducted on the genetics of male infertility, ~60–75% of infertile male patients appear to be idiopathic. This is matched with the number of men visiting *in vitro* fertilization clinics; nearly 60% have DNA damage and 80% of these men are found to have idiopathic causes beyond diagnosis confirmed by established standard testing protocols.^14^ Based on investigations carried out using different platforms including biochemical, genetic and molecular studies it could be inferred that male infertility is a complex heterogeneous multifactorial disorder, which has met with limited success in identifying the real causes behind this condition.^8^ A wide spectrum of tests for genetic as well as epigenetic factors ranging from SNPs, variable number tandem repeats and copy number variations identified using array-based profiling techniques were performed to identify diseased IMI genes.^6^ These genes were postulated to play diverse roles in the complex process of packaging a disease-free male gamete. High-risk genes mostly identified include Y chromosome-linked genes, X-linked candidate genes and the deleted in azoospermia-like (*DAZL*) gene, which is an autosomal homologue of the Y-chromosomal *deleted in azoospermia *(*DAZ*) gene cluster.^15^

With genomics taking the center stage following whole-genome sequencing (WGS), thanks to next-generation sequencing (NGS) becoming a benchmark for disease diagnosis as more bench side discoveries are coming to the mainstream exploiting the aberrations in the structural gene changes associated with various diseases.^16^ More data are being generated, which form a rich source for data miners for investigating novel gene−disease associations.^17–20^ Most disease phenotypes result from accumulation of several genomic alterations resulting in sub-genotypes, which becomes part of a complex network. Prior knowledge on the causative genes and the underlying pathways they are associated with is a prerequisite to dwell deeper into the molecular basis associated with a pathological phenotype as complex as IMI, considering a larger part of the interactions leading to this disease is still ambiguous.^21^ The success of the tests will depend on functional gene evidence, using which a disease condition or blockage of vital cell functions such as metabolism, cell cycle and structural integrity of the cells could be explained with proper validation.^22^

With more databases beginning open access it gives an opportunity to discover novel gene associations and provides the ideal opportunity to discover more of their translation effects in mediating disease progression.^21,23^ However, a large void exists between the genomics-based discoveries and successful identification of protein-based markers considering the fact that not all genes could successfully translate into proteins. Only a small percentage of disease mutations find their way in causing altered protein expression through protein misfolding as well as structural changes in their binding sites of the genomic elements such as transcription factors and small RNAs.

The current study identifies all the genes reported so far as associated with IMI and tries to build meaningful association between key regulators of genes correlated with infertility, such as ROS, and oxidative stress (OS) genes. SNPs associated with candidate target genes were further subjected to in-depth analyses to identify their functional roles in effecting IMI phenotype.

## Results

**Figure 1:**
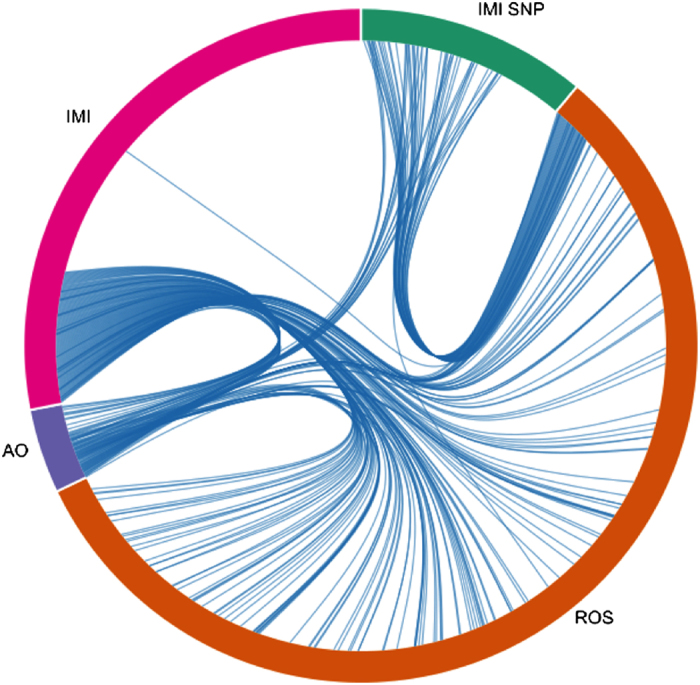
The figure depicts the shared or common genes between four gene sets that are associated with IMI: (1) 484 genes associated with IMI (IMI) (with no SNP associated with it); (2) 192 IMI genes with SNPs; (3) 981 ROS genes; and (4) 70 antioxidant genes (AO).

Through data mining and manual curation we catalogued the following set of genes related to IMI: (1) 484 genes associated with IMI (with no SNP associated with it); (2) 192 genes with SNPs; (3) ROS 981 genes; and (4) 70 AO genes. In our *in silico* analysis we generated a list of genes associated with (1) IMI, (2) those associated with ROS and (3) AO genes. We then matched them with SNPs previously reported in these set of genes. The 192 genes carrying SNPs were filtered from ‘SNPs3D disease candidate gene database’ specific to IMI and the selection criterion was that these genes were carrying either one or more than one SNP—reported for IMI. The 484 genes were identified for IMI; however, there were no reports of SNPs associated with these set of genes. Similarly, for both ROS and AO genes, the presence of SNPs was verified using SNPs3D disease candidate gene database. ROS is a key physiological determinant and major contributing factor identified as a leading cause for the progression of IMI. The effects were mainly targeted towards DNA damage, leading to a spectrum of abnormalities associated with sperm head morphology and impaired motility. While AO act as protective in nature from the deleterious effects of ROS, imbalance between ROS and AO results in DNA damage and contributes to the progression of male infertility. Only a fraction of the SNPs containing genes were associated with the number of genes reported for IMI. Presence of SNPs in any of the IMI genes as well as the ROS and AO genes could affect the normal functioning of these genes. With this in mind, we selected the genes with and without SNPs and performed the functional analysis to identify their roles. It is important to identify gene pairs associated with IMI and genes with known SNPs associated with IMI, since any change in the gene structure in the SNP encoding genes in the affected population could render them more susceptible to IMI; the same applies for ROS and AO genes (Figure 1). In addition to the 672 genes, defective genes associated with spermatogenesis for spermatozoa (114 genes) and spermatocytes (72) from other animal models, which will be useful to study human associated IMI (data not included), were catalogued.

Cellular localization studies showed bulk of the genes associated with IMI were membrane associated (plasma membrane and membrane parts), indicating that the damage caused in IMI could be traced to membrane-associated functions and possible structural failure in the spermatozoa membrane in IMI individuals (Supplementary Figure 1). This correlates with the influence of ROS in damaging DNA and telomeres in the spermatozoid, as well as increased ROS in seminal plasma, which could damage the spermatozoa membrane. Gene set analysis showed specific subsets of gene families associated with IMI were involved with transcription factors, cytokines and growth factors, and cell differentiation markers (Supplementary Figure 2). Transcription factors (TFs) could influence a large, diverse set of genes in the cell. The other classes of genes are cytokines and growth factors, and cell differentiation markers. These sets of genes could play a key role in the signal transduction mechanisms associated with spermatogenesis.

**Figure 2:**
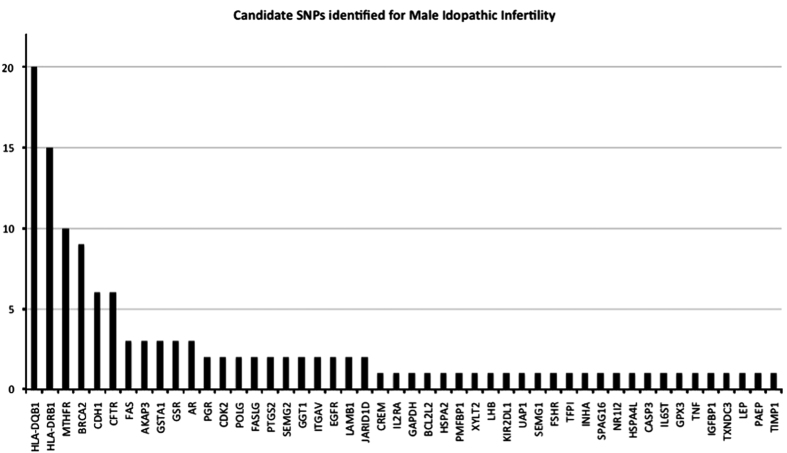
Candidate SNPs identified for IMI and their frequency distribution across different genes implicated with the disease. *HLA-DQB1, HLA-DRB1, MTHFR, BRCA2, CDH1 *and* CFTR* genes inherited more than five SNPs or more associated with IMI.

Through mining approaches and manual curation we identified a total of 559 genes harboring SNPs associated with male infertility. Of these 106 genes were found to harbor one or more number of SNPs associated with male infertility (Figure 2). Functional analysis revealed that associated genes carry a wide array of essential functions associated with spermatogenesis (Supplementary Table 1). Among the 981 ROS genes, 37 genes for IMI-encoding SNPs were identified using *in silico* analysis (Supplementary Figure 3). Network analysis identified key genes that are central to the ROS-mediated IMI.

Gene clusters corresponding to 672 genes (IMI-specific genes) were curated and subjected to downstream analysis. The foremost active gene sets were associated with (1) apoptosis, (2) spermatogenesis and (3) ROS in effecting the IMI (Table 1). In addition, cytokine signaling, estrogen response and xenobiotic metabolism were among the top affected listed in IMI. The study is in accordance with the known disease pathways affecting both spermatozoa and seminal plasma in infertile patients. However, epithelial mesenchymal transition (EMT), inflammatory response and allograft rejection were unique to newly identified list of factors, which could result in IMI.

Y-chromosome genes were found to play a key role by harboring several clusters of genes critical to spermatogenesis. The Y-chromosome cluster genes formed the majority (of the top 100 genes) of the clusters identified in IMI (Supplementary Figure 4). These could provide vital clues for identifying the underlying hidden causes for this disease condition. The major Y-chromosome proteins include the testis-specific human protein RBMY, a probable human spermatogenesis factor protein, RPS4Y2, PRY2, XKRY2, VCY1B, VCY, UTY, USP9Y, testis-specific transcript, Y-linked 9A (TTTY9A), TMSB4Y, DDX3Y, deleted in azoospermia 1, deleted in azoospermia 3, deleted in azoospermia 2, deleted in azoospermia 4, chromodomain protein, Y-linked, 2A (CDY2A), chromodomain protein, Y-linked, 2B (CDY2B), B, CDY1B, CDY1, BPY2C, BPY2, ASSP6, EIF1A, JARID1D, heat shock transcription factor, Y linked 2 (HSFY2) and GOLGA2LY1. Genes identified in this cluster are specific to Y chromosome and some of these genes could play a novel role in contributing to the pathogenicity of IMI. Modular analysis showed presence of six modules comprising the 34 genes located in the Y chromosome (Supplementary Figure 5). The core genes were *ubiquitin C* (*UBC*), *DAZ1*, *DEAD* (Asp−Glu−Ala−Asp) *box helicase 3* and *Y-linked* (DDX3Y). We are currently investigating the role of these core genes in mediating other Y chromosome genes associated with spermatogenesis.

**Figure 3:**
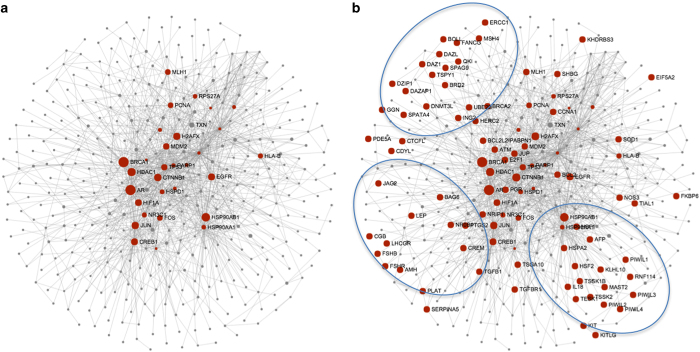
Representation of gene network clusters associated with gamete generation in IMI (**b**). (**a**) The background core gene set network. Three noticeable clusters were precocious and located in the periphery of the gene network.

Modular analysis of genes identified in Y-chromosome showed the presence of six modules involving 34 genes located in the Y chromosome (Supplementary Figure 5b). Analysis of Y chromosome genes also showed modules specific for gametogenesis as identified through *in silico* analysis (Supplementary Figure 5C). Further, these gene clusters will help to create relationships between structural gamete generation genes and core genes. Mapping of IMI genes associated with gamete generation showed their distribution in the peripheral region of the network (Figures 3a and b). Three distinct clusters were precocious and located in the periphery of the gene network. The results indicate the heterogeneous nature of the genes and pathways associated with gamete generation. The core genes located to the center of the network could exert a major influence in the regulation of gametogenesis.

**Figure 4:**
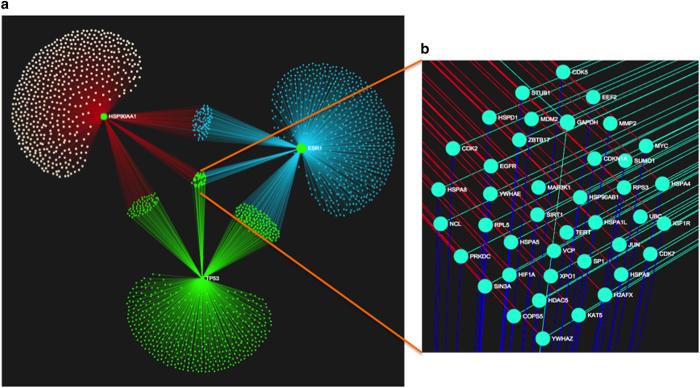
Graphical representation of network clusters formed by three core genes *TP53*, *HSP90AA1* and *ESR1* identified from 16 SNPs carrying antioxidant genes associated with IMI. These three genes control a larger network of 1835 genes connected by a total of 2179 edges (**a**). Structural alterations in any one of these genes could result in severe imbalances, taking into consideration the large number of genes they influence. Sets of genes under the control of three core genes (**b**) are highlighted as a projection from the central core network.

AO genes play a protective role in preventing oxidation-induced damages in the testicular microenvironment. We identified a total of 70 AO genes, of which 16 (Supplementary Figure 6) have known SNPs associated with it and has association with IMI (Figure 4). SNPs associated with AO genes could severely compromise in their protective function during spermatogenesis. Further analysis of these genes showed a central cluster comprising of three genes, *TP53*, *HSP90AA1* and *ESR1*, which controls a total number of 1835 genes (Figure 4b). Forty one genes were common to the three core genes which includes genes such as *TERT*, *MYC*, *UBC*, *MAP3K1* and* SP1*. Association analysis between key nodes in the network showed that the three genes *TP53*, *HSP90AA1* and *ESR1* are interlinked and are responsible for several biological process associated with spermatogenesis (Supplementary Table 3).

Matching of results from the current study with previously published MS results showed unique distribution of genes across the spermatozoa and seminal plasma (Supplementary Figures 7A–C). Comparison with previously reported proteins associated with spermatozoid^24^ and seminal plasma^25^ under high, medium and low ROS conditions and those identified in the current study using *in silico* approaches identified protein coding 20 genes corresponding to spermatozoa as well as 34 genes corresponding to seminal plasma, respectively, that were not previously associated with IMI (Supplementary Figure 8; Supplementary Table 3). Functional analysis using the above set of genes corresponding to spermatozoa as well as seminal plasma showed unique functions associated to each category that correlated with their location (Tables 2a and 2b). The study also identified unique functions previously not encompassed by other studies for genes and gene products and proteins identified in spermatozoa as well as seminal plasma (Tables 2a and 2b).

## Discussion

We have performed a comprehensive analysis on the role of potential faulty genes associated with key determinants affecting IMI. Towards this, we have used a suite of techniques to populate as well as evaluate these genes and assign them to different networks and pathways that could relate to IMI. Bulk of the genes associated with IMI were related to ROS, which could explain the role of imbalance in ROS genes and their protein products present in seminal plasma as well as spermatozoa in inflicting damage on plasma membrane and membrane parts of spermatozoa, which could possibly explain the primary cause for structural malformation and impeded motility that have been considered as the primary causes leading to the male infertility phenotype.

ROS genes numbering to about 1000 constituted the bulk of the genes associated with IMI. Hence the role of ROS in infertility attains much significance. Results from the current study correlate with the influence of ROS in damaging DNA and telomeres in the spermatozoid as well as increased ROS in seminal plasma, which could damage the spermatozoa membrane. The oxidative equilibrium of cells in the testicular microenvironment is critical for the normal spermatogenic process. Results from the study showed SP1 transcription and interlinks of the ubiquitin gene *UBC* with the ROS-associated genes such as apoptosis-related cysteine protease *caspase 3* (*CASP3*) and other nine genes, *tumour necrosis factor* (*TNF*)*-alpha*, *super oxide dismutase* (*SOD*) and *nitric oxide synthase* (*NOS3*). These set of genes are pivotal to the oxidative equilibrium of cells in the testicular microenvironment. Studies have shown the presence of deleterious SNPs in the *CASP3* gene could explain the instability associated with male infertility. From the results it is clear that *CASP3*, which interacts with caspase-8 and caspase-9, plays a central role in mediating ROS in IMI.

Genetic factors contribute up to 15–30% cases of male infertility. Our analysis shows IMI genes coding for transcription factors and growth factors form the major category, followed by cell differentiation and protein kinase genes. Transcription factors have multiple roles in the signaling mechanisms associated with spermatogenesis. Only few studies were performed in deciphering the role of TFs in effecting male infertility.^26–29^ A-MYB (MYBL1) transcription factor is a master regulator of male meiosis^26^ and it is an integral part of the cell cycle. *Sex-determining region Y* (*SRY*) gene expresses a TF that switches on the genes for male sexual differentiation.^30^ Studies involving TFs were mostly carried out in animal models^27–29^ and could possibly indicate a similar role in humans. Aberrant binding of TFs in mutated/SNPs in gene promoters or deregulation in TFs due to inherent nucleotide changes could render them ineffective, thus compromising on the process of spermatogenesis.

Occurrence of a large number of SNPs in key genes associated with spermatogenesis indicates their essential role in male gametogenesis. We have shown key genes *HLA-DQB1*,* HLA-DRB1*,* MTHFR*,* BRCA2*,* CDH1* and* CFTR* inherit five or more SNPs associated with IMI. Most SNP-populated genes related to male infertility include *HLA-DQB1* (20 SNPs), *HLA-DRB1* (15 SNPs), *MTHFR* (10 SNPs), *BRCA2* (9 SNPs), *ACE* (8 SNPs), *CFTR* (6 SNPs), *CDH1* (6 SNPs), *SRD5A2* (6 SNPs), *HLA-DQA1* (5 SNPs) and *MMP9* (5 SNPs). These genes perform several vital functions associated with sperm development and further downstream functions. HLA class II genes may influence spermatogenesis and male gamete function,^31^ while mutations in *MTHFR*,^32^ *BRCA2*,^33^ *CDH1* and *CFTR* were known to play key roles in normal spermatogenesis. Previous reports have shown both *HLA-DQB1* and *HLA-DRB1* could influence the reproductive process, which could mainly affect the gamete quality and embryonic development.^31^ It is found that in severe cases of male infertility the above HLA loci have significantly different allele frequencies when compared to males with normozoospermia. The changes in the allele frequencies in these genes could also influence other genes in the near vicinity, which might be involved in spermatogenesis and male gamete function. Transport of Cl(−) and HCO(3)(−) through cAMP-activated channels will have a major role in male infertility for processes such as capacitation.^34^ The CFTR gene, the *cystic fibrosis transmembrane conductance regulator* (*CFTR*), is a cAMP-activated Cl(−) and HCO(3)(−) conducting channel and has been investigated to study the effect of mutations in this gene and its role in male infertility. Studies show *CFTR* could play a major role in sperm capacitation by mediating HCO(3)(−) entry that is essential for capacitation. Evidence from clinical studies has shown increased mutation frequency or reduced CFTR expression in men with congenital bilateral absence of vas deferens or sperm abnormalities, such as azoospermia teratozoospermia and oligoasthenospermia. Studies on Sertoli cells and germ cells, as well as testes from CFTR knockout mice or a cryptorchidism model, indicate the involvement of CFTR in spermatogenesis through the HCO(3)(−)/sAC/cAMP/CREB(CREM) pathway and the NF-kappaB/COX-2/PGE(2) pathway.^34^ Studies on male infertility have mostly focused on studying the effect of the allele and genotype frequencies on polymorphisms of key genes that were identified to play a major role in various disease conditions associated with male infertility, such as azoospermia or severe oligozoospermia. Two such genes that were found in the current study were *MTFHR* and *BRCA2*. Epigenetics is an emerging area in the field of infertility research. Genes associated with the folate metabolism have been extensively studied in relation to infertility. One of the key genes in this pathway is* methylenetetrahydrofolate reductase* (*MTFHR*), which plays a key role in DNA synthesis and remethylation reactions. Polymorphism associated with MTFHR gene has been associated with male infertility.^35^ Patients with severe oligozoospermia showed statistically significant correlation with the homozygous (C/C) A1298C polymorphism of the *MTHFR* gene. This is a key observation since defects in DNA synthesis or methylation of genes associated with spermatogenesis pathway will have a major impact on the processing of spermatids and mature spermatozoa. Similarly, for *BRCA2* gene the common SNP, N372H, in human *breast cancer susceptibility gene 2* (*BRCA2*) was significantly increased in patients with IMI,^33^ and in patients with azoospermia or severe oligozoospermia. Studies on spermatogenic stem cells as well as germ cells are an evolving field to find cure for many of the aberrations associated with defective spermatogenesis. CDH1, previously known as E-cadherin, is one of the genes identified by our study to have been previously associated with the undifferentiated type A spermatogonia when tested in mouse models.^36^ CDH1 is a marker for stem cells. Positive staining for CDH1 could help in isolating these populations of stem cells in human samples and may help in the regenerative medicine field associated with reproductive medicine. In another study the CDH1 part of APC/C(FZR1) was found to be an essential regulator of spermatogonial proliferation of germ cells.^37^ Hence CDH1 could play a major role during the early events associated with spermatogenesis.

A known SNP identified in jumonji, AT rich interactive domain 1D (JARID1D), encodes a protein-containing zinc finger domain. A short peptide derived from this protein is a minor histocompatibility antigen, which can lead to graft rejection of male donor cells. SNP distribution across genes could play a key role in IMI.^38^ We looked at the distribution of SNPs among the diseased genes associated with different processes associated to male reproduction. The major process affected by SNPs includes spermatogenesis, which is caused by 141 genes with 1871 SNPs, followed by sperm motility (17 genes with 503 SNPs), spermatid development (8 genes with 29 SNPs), binding of sperm to zona pellucida (9 genes with 162 SNPs) and fusion of sperm to egg plasma membrane (6 genes with 25 SNPs). The SNP distribution among the critical genes associated with IMI is a strong evidence to show the role of these genetic alterations in causing abnormal gene regulation in critical genes associated with spermatogenesis.

Genomic heterogeneity and instability of the human Y chromosome in male factor infertility are well documented.^39–41^ We have shown that the Y chromosome harbors clusters of genes, which are associated with IMI. Two loci were found to harbor the pathogenic gene *Chromosome Y cytob*; chryq11 and chryp11 harbor the maximum number of genes associated with the disease condition, followed by chr6p21. Six distinct modules were associated with Y-chromosome genes; these modules code for a variety of functions including gamete generation, which are distributed mostly in the periphery of the core genes and found in clusters, indicating that assigned roles for each cluster and any aberrations in core genes will have a major impact on the stability of these genes and might lead to infertility. Core genes controlling these modules include (1) *DAZ1* (Chr Y), (2) *UBC *(Chr12), (3) *DEAD* (Asp−Glu−Ala−Asp) *box helicase 3 *(Chr Y) and (4) *Y-linked* (DDX3Y) (Chr Y), which are all the major genes coded by Y-chromosome genes,^39,42–44^ except *UBC,* which is a chromosome 12 gene.

The AO class of genes performs a wide array of functions associated with homeostasis during spermatogenesis by providing a conducive environment for the gamete generation process both in the testicular microenvironment as well as in the seminal plasma.^45^ The major role of these genes is to protect the male gamete from the deleterious effects of ROS end products. Imbalance in these genes leads to severe burden in their protective role and compromises the stability of both spermatozoa as well as seminal plasma.^46^ The prominent roles of the genes in this category are related to nitric oxide (eNOS) metabolism, stabilization of p53 and G1−G2 mitotic transitions; the biological processes involving AO genes include mitochondrial membrane stability, a key to the energy metabolism of spermatozoids, catalase activity for normal scavenging of free radicals and the intrinsic apoptotic signaling pathway in response to DNA damage (see Supplementary Table 2). TP53, HSP90AA1 and ESR1 were found to be the master regulator of these sets of functions associated with AO genes, again strengthening the role of TFs in normal spermatogenesis events.

Genes and associated proteins detected in seminal plasma as well as spermatozoa presented a unique picture. The functional roles assigned to these genes as well as proteins matched their biological location and could also explain the possible mechanisms related to the damage associated with the process of spermatogenesis. Functional enrichment analysis of unique genes corresponding to spermatozoa showed that genes and proteins related to EMT, estrogen response, novel HEDGEHOG signaling genes, apoptosis and xenobiotic metabolisms were key targets for IMI-specific genes. This is in accordance with previous studies,^47^ which have reported ROS-induced apoptosis^48^ and aberration in xenobiotic metabolism^49^ as markers of progression of pathogenesis associated with male infertility. However, EMT and HEDGEHOG signaling genes have to be investigated to elucidate their roles in IMI. (They could have an indirect effect; for example, Wt1 deletion in mouse may affect the EMT and gonad formation^50^ might consequently affect the spermatogenesis.) A previous study by Szczepny *et al.*^51^ reported deletions in any one of the HEDGEHOG signaling genes cause infertility. VEGFA and PLG were reported as transcription stimulators and could be involved in mediating an array of genes associated with spermatogenesis.^52^ Proteins unique to seminal plasma showed a different composition to the one observed for spermatozoa. A total of 34 genes/proteins were found to be associated with oxidative phosphorylation, ROS and spermatogenesis. This is likely to help identify gene products associated with ROS and apoptosis, since they could be leftovers from the cells undergoing cell death triggered by an abnormal ROS environment in the affected cells responsible for defective spermatogenesis. Other than the above candidate genes, gene sets associated with MYC targets and adipogenesis were also detected in seminal plasma. The source of these proteins could be traced back to the corresponding genes in tissues secreting them, or the genes might be released into the seminal plasma through death signaling or other mechanisms, which remains to be investigated.

There is a huge potential to translate the proteins and associated genes that were identified in the current study. Genes and proteins need to be tested in patient samples during each clinical diagnosis of male infertility. A panel of markers could be created and multiplex approaches as well as NGS gene panels constructed to test the efficacy of these markers in a larger cohort of patients in the general population.

The study has used data from published articles related to IMI. We have proposed few targets in our current study that will have significant effect on the research in the male infertility and on understanding the pathophysiology of the disease. The current study used literature search based on only published data or the information currently available in the literature. Some of the results from the current study could be matched with data output from mass spectrometry studies related to the disease condition, as we have shown for certain marker proteins for spermatozoa and seminal plasma. Hence future efforts should be focused on understanding the biological relevance as well as the real expression patterns of these genes in the tissues and body fluids (seminal plasma) and the role they play in the bigger picture under each specific disease condition. Curing male factor infertility is a major challenge, which is compounded by the multiple factors associated with this disease. Reversal of male infertility depends on a host of biochemical, physiological as well as genetic factors, which requires an integrated approach to address the limitations of each strategy. Significant amount of bench work has already been done in the field of male infertility research; however, very few attempts were carried out to bring all the research under one platform. Using computation biology approaches, meaningful results could be drawn from multiple experiments conducted at different laboratories. Solutions could be provided for most of the issues concerning male infertility by identifying the basic underlying phenomena at the pathophysiological levels caused by impaired genetic mechanisms, which could be rectified through proper interventions. The advent of OMICS field has helped to reduce this complexity to some extent by providing a global landscape for this unique pathogenic condition. The challenge now will be to approach the three OMICS platforms, namely gene expression at the level of mRNA, proteomes and metabolome, to identify key nodes that are crucial for the process of spermatogenesis. The coming years will see lot of research in these three areas to give a better treatment option for patients with IMI.

In conclusion, this study has identified genes and possibly the proteins they might code for, which could play central roles in the pathogenesis of IMI. Pathogenic SNPs present in genes coding for ROS function, AO genes and genes associated with IMI could help to narrow down target genes as well as identify novel regulator genes that could help to understand the underlying networks that are associated with the bigger picture of pathogenesis associated with IMI. Moreover, the identification of enriched genes, pathways, gene modules and clusters as well as intermediate regulators could shed more information on the global network of different pathways related to this heterogeneous disease condition. Such an in-depth understanding of the individual components as well as the functions associated with it could help to detect as well as monitor the disease at an early stage and could lead to development of new treatment modalities that could help in the reversal of certain types of IMI. Finally, through the process of *in silico* approaches as well as data-mining strategies it is possible to identify pre-existing relationships as well as novel associations previously not described and worth investigating in the future. It remains to be seen how proteins coded by these genes could actually be detected to play an effective role in causing the diseased IMI phenotype.

## Materials and methods

We used a suite of both computational as well as manual data-mining techniques to index genes associated with IMI. Genes associated with IMI, ROS genes implicated with male infertility, AO genes and genes affected by SNPs, and known roles in IMI were curated both manually as well as through computation approaches. Data mining was performed using online PubMed searches on publications referring to IMI. The data pertaining to the current study were obtained from PubMed for January−March 2016. The sources of genes for the current study were obtained as follows: extensive data mining was carried using computational and manual approaches. Each article was thoroughly searched for genes reported/associated with each condition that was used for the current study, and a combined list was created using this approach. Microsoft Excel was used to search for duplicated gene names. PubMed, SCOPUS, MEDLINE and other publically available databases were used for the current study. Standard online VENN diagram tools were used to create VENN diagrams as well as identify overlapping genes. These curated and annotated genes were further used for downstream functional analysis. Genes associated with IMI, ROS genes implicated with male infertility, AO genes and genes affected by SNPs, and known roles in IMI were curated both manually as well as from SNPs3D disease candidate gene database. A suite of publically available software such as Cytoscape and other visualization platforms were used to create meaningful functional networks. For functional annotation and enrichment analysis we used publicly available bioinformatics annotation tools and databases such as GO Term Finder, GO Term Mapper, UniProt, Software Tools for Researching Annotations of Proteins (STRAP)^53^ and Database for Annotation, Visualization and Integrated Discovery (DAVID) (http://david.niaid.nih.gov)^54^ to obtain a consensus-based, comprehensive functional context for the large list of genes and proteins derived from the datamining approach. Further gene associations and functional gene set enrichments were performed to create networks associated with functional relevance such as biological processes, organelle structure and molecular pathways using the Molecular Signatures Database (MSigDB). The results from the current study were matched with wet lab studies previously conducted in our laboratory using mass spectrometry studies to identify unique candidate markers for both spermatozoa^24^ and seminal plasma.^25^

## Figures and Tables

**Table 1:** Key functional roles identified by analysis of 672 genes associated with idiopathic male infertility, which match the pathophysiological phenotype of molecular interactions associated with idiopathic male infertility

	*Gene set name*	*No. of genes in the gene set (*K*)*	*Description*	P*-value*
1	**APOPTOSIS**	**161**	**Genes mediating programmed cell death (apoptosis) by activation of caspases**	**3.28E−28**
2	**SPERMATOGENESIS**	**135**	**Genes upregulated during production of male gametes (sperm), as in spermatogenesis**	**1.43E−25**
3	ALLOGRAFT REJECTION	200	Genes upregulated during transplant rejection	5.18E**−**25
4	IL6 JAK STAT3 SIGNALING	87	Genes upregulated by IL6 [GeneID=3569] via STAT3 [GeneID=6774], e.g., during acute phase response	4.45E**−**16
5	**REACTIVE OXYGEN SPECIES PATHWAY**	**49**	**Genes upregulated by reactive oxigen species (ROS)**	**1.70E−13**
6	EPITHELIAL MESENCHYMAL TRANSITION	200	Genes defining epithelial−mesenchymal transition, as in wound healing, fibrosis and metastasis	2.31E**−**13
7	ESTROGEN RESPONSE LATE	200	Genes defining late response to estrogen	2.31E**−**13
8	XENOBIOTIC METABOLISM	200	Genes encoding proteins involved in processing of drugs and other xenobiotics	2.01E**−**12
9	INFLAMMATORY RESPONSE	200	Genes defining inflammatory response	1.66E**−**11
10	P53 PATHWAY	200	Genes involved in p53 pathways and networks	1.66E**−**11

Key events such as apoptosis, defective spermatogenesis and reactive oxygen species (shown in bold) are the hallmarks for this disease condition.

**Table 2:** Functional analysis showing genes associated with sperms' 20 genes are involved with EMT, xenobiotic metabolism, apoptosis estrogen response and KRAS signaling, which matches with the pathological phenotype of IMI

	*Gene set (sperm associated)*	*No. of genes*	*Genes*	P*-value*
1	EPITHELIAL MESENCHYMAL TRANSITION	4	IGFBP2, IGFBP4, VEGFA, TGFB1	1.60E**−**06
2	UV RESPONSE UP	3	IGFBP2, IL6ST, GPX3	4.35E**−**05
3	XENOBIOTIC METABOLISM	3	IGFBP4, PLG, JUP	8.76E**−**05
4	HEDGEHOG SIGNALING	2	VEGFA, PLG	1.12E**−**04
5	IL6 JAK STAT3 SIGNALING	2	TGFB1, IL6ST	6.58E**−**04
6	APOPTOSIS	2	GPX3, TIMP2	2.22E**−**03
7	COMPLEMENT	2	PLG, TIMP2	3.40E**−**03
8	ESTROGEN RESPONSE EARLY	2	IGFBP4, IL6ST	3.40E**−**03
9	ESTROGEN RESPONSE LATE	2	IGFBP4, IL6ST	3.40E**−**03
10	KRAS SIGNALING DN	2	IGFBP2, SEPP1	3.40E**−**03

**Table 3:** Seminal plasma genes and proteins presenting a contrasting picture when compared to proteins and their functions from spermatozoa

	*Gene set (seminal plasma associated)*	*No. of genes*	*Genes*	P*-value*
1	OXIDATIVE PHOSPHORYLATION	6	SLC25A3, VDAC1, GPX4, SDHB, VDAC2	7.66E**−**09
2	MYC TARGETS V1	5	VDAC3, SLC25A3, VDAC1, RPS5, C1QBP	3.73E**−**07
3	REACTIVE OXIGEN SPECIES PATHWAY	3	VDAC3, GPX4, CAT, PRNP	6.66E**−**06
4	ADIPOGENESIS	4	GPX4, SDHB,	1.46E**−**05
5	SPERMATOGENESIS	2	VDAC3, ART3, CAT, ATP1B3	4.52E**−**03

The 34 genes were associated with previously known functions such as oxidative phosphorylation, ROS and spermatogenesis. The new set of functions involving MYC targets V1 and adipogenesis are reported for the first time and requires further in-depth investigations to establish their roles in defective spermatogenesis leading to IMI.
