# Supplementary material for: Pathogenic landscape of idiopathic male infertility: new insight towards its regulatory networks
Source: NPJ Genom Med. 2016 Aug 17;1:16023–. doi: 10.1038/npjgenmed.2016.23 (PMC5685305; doi:10.1038/npjgenmed.2016.23)
Supplement: Supplementary Table 1 [file npjgenmed201623-s1.doc]

| **Supplementary Table 1.** SNP distribution across genes associated with spermatogenesis. From the table it is clear that SNPs are distributed across most of the critical genes that play a crucial role in normal gametogenesis. | | | |
| --- | --- | --- | --- |
|  | **GO: Biological Process** | **#Genes** | **#SNPs** |
|  | **Name** |
| 1 | Sperm axoneme assembly | 1 | 4 |
| 2 | Sperm capacitation | 1 | 2 |
| 3 | Sperm chromatin condensation | 1 | 3 |
| 4 | Sperm displacement | 1 | 3 |
| 5 | Sperm ejaculation | 1 | 2 |
| 6 | Sperm mitochondrion organization and biogenesis | 1 | 2 |
| 7 | Sperm motility | 17 | 503 |
| 8 | Spermatid development | 8 | 29 |
| 9 | Spermatid differentiation | 1 | 8 |
| 10 | Spermatid nuclear elongation | 1 | 3 |
| 11 | Spermatogenesis | 141 | 1871 |
| 12 | Spermidine biosynthetic process | 3 | 25 |
| 13 | Spermidine catabolic process to deoxyhypusine, using deoxyhypusine synthase | 1 | 12 |
| 14 | Spermine biosynthetic process | 1 | 20 |
| 15 | regulation of fusion of sperm to egg plasma membrane | 1 | 7 |
| 16 | Fusion of sperm to egg plasma membrane | 6 | 25 |
| 17 | Binding of sperm to zona pellucida | 9 | 162 |
