# Supplementary material for: Pathogenic landscape of idiopathic male infertility: new insight towards its regulatory networks
Source: NPJ Genom Med. 2016 Aug 17;1:16023–. doi: 10.1038/npjgenmed.2016.23 (PMC5685305; doi:10.1038/npjgenmed.2016.23)
Supplement: Supplementary Table 2 [file npjgenmed201623-s2.doc]

| **Supplementary Table 2**. Reactome analysis of 16 antioxidant genes showed the genes were involved with eNOS activation, cell cycle checkpoints, and mitosis. While pathways affected included mitochondria associated pathways such as those involved with mitochondrial membrane organization, transport and organization. The outcome from this study is in accord with the role of mitochondria in effecting homeostasis in antioxidant metabolism. | | | | | |
| --- | --- | --- | --- | --- | --- |
|  | **REACTOME ANALYSIS PATHWAY** |  |  | **BIOLOGICAL PROCESS PATHWAY** |  |
|  | Pathway | P. Value |  | Pathway | P. Value |
| 1 | eNOS activation | 0.00404 | 1 | mitochondrial membrane organization | 3.17E-05 |
| 2 | Tetrahydrobiopterin (BH4) synthesis, recycling, salvage and regulation | 0.00583 | 2 | mitochondrial transport | 0.000366 |
| 3 | Sema3A PAK dependent Axon repulsion | 0.00717 | 3 | protein import | 0.00107 |
| 4 | Signaling by constitutively active EGFR | 0.00851 | 4 | mitochondrion organization | 0.00135 |
| 5 | Metabolism of nitric oxide | 0.00896 | 5 | regulation of catalytic activity | 0.00178 |
| 6 | eNOS activation and regulation | 0.00896 | 6 | cell development | 0.00215 |
| 7 | Cell Cycle | 0.0165 | 7 | interphase of mitotic cell cycle | 0.00272 |
| 8 | Nuclear signaling by ERBB4 | 0.0183 | 8 | interphase | 0.00282 |
| 9 | Autodegradation of the E3 ubiquitin ligase COP1 | 0.0236 | 9 | anatomical structure formation involved in morphogenesis | 0.00313 |
| 10 | Nuclear Receptor transcription pathway | 0.0236 | 10 | cellular membrane organization | 0.00318 |
| 11 | Stabilization of p53 | 0.0241 | 11 | regulation of molecular function | 0.00389 |
| 12 | p53-Dependent G1/S DNA damage checkpoint | 0.0263 | 12 | protein targeting | 0.00425 |
| 13 | p53-Dependent G1 DNA Damage Response | 0.0263 | 13 | response to organic substance | 0.00535 |
| 14 | G1/S DNA Damage Checkpoints | 0.0276 | 14 | positive regulation of cellular metabolic process | 0.00553 |
| 15 | Regulation of actin dynamics for phagocytic cup formation | 0.0276 | 15 | positive regulation of metabolic process | 0.00668 |
| 16 | Loss of proteins required for interphase microtubule organizationí‰Œæfrom the centrosome | 0.0289 | 16 | anatomical structure morphogenesis | 0.00764 |
| 17 | Loss of Nlp from mitotic centrosomes | 0.0289 | 17 | protein import into nucleus, translocation | 0.00817 |
| 18 | Semaphorin interactions | 0.032 | 18 | negative regulation of DNA replication | 0.00879 |
| 19 | Centrosome maturation | 0.0382 | 19 | negative regulation of signal transduction | 0.00882 |
| 20 | Recruitment of mitotic centrosome proteins and complexes | 0.0382 | 20 | intracellular protein transport | 0.00889 |
| 21 | Fcgamma receptor (FCGR) dependent phagocytosis | 0.0382 | 21 | positive regulation of transcription from RNA polymerase II promoter | 0.00904 |
| 22 | G2/M Transition | 0.0451 | 22 | base-excision repair | 0.00942 |
| 23 | Mitotic G2-G2/M phases | 0.0464 | 23 | growth | 0.00993 |
|  |  |  | 24 | protein complex assembly | 0.0104 |
|  |  |  | 25 | intrinsic apoptotic signaling pathway in response to DNA damage | 0.0107 |
