# Supplementary material for: Pathogenic landscape of idiopathic male infertility: new insight towards its regulatory networks
Source: NPJ Genom Med. 2016 Aug 17;1:16023–. doi: 10.1038/npjgenmed.2016.23 (PMC5685305; doi:10.1038/npjgenmed.2016.23)
Supplement: Supplementary Table 3 [file npjgenmed201623-s3.doc]

| **Supplementary Table 3**. List of genes unique to spermatozoid (20 genes: Table 6) and seminal plasma (34 genes: Table 6) associated with idiopathic male infertility previously not reported in other studies. | | | |
| --- | --- | --- | --- |
|  | **Sperm unique IMI genes** |  | **Seminal plasma unique IMI genes** |
| 1 | DDR1 | 1 | ART3 |
| 2 | EIF5A2 | 2 | VDAC1 |
| 3 | FCGBP | 3 | VDAC3 |
| 4 | GPX3 | 4 | HIST1H2AA |
| 5 | GSTT1 | 5 | PTGS2 |
| 6 | HBB | 6 | SPAM1 |
| 7 | IGFBP2 | 7 | H2AFX |
| 8 | IGFBP4 | 8 | CD46 |
| 9 | IGFBP5 | 9 | C1QBP |
| 10 | IL6ST | 10 | ATP1B3 |
| 11 | JUP | 11 | VDAC2 |
| 12 | LCN1 | 12 | SDHB |
| 13 | NEU1 | 13 | PRSS21 |
| 14 | PLG | 14 | PRL |
| 15 | PRSS1 | 15 | CHDH |
| 16 | RPS27A | 16 | GPX4 |
| 17 | SEPP1 | 17 | ACTC1 |
| 18 | TGFB1 | 18 | AKAP3 |
| 19 | TIMP2 | 19 | HIST2H2AC |
| 20 | VEGFA | 20 | CAT |
|  |  | 21 | DYNLT1 |
|  |  | 22 | HLA-DRB1 |
|  |  | 23 | PRNP |
|  |  | 24 | RPL11 |
|  |  | 25 | RPL19 |
|  |  | 26 | RPL24 |
|  |  | 27 | RPL30 |
|  |  | 28 | RPL4 |
|  |  | 29 | RPS13 |
|  |  | 30 | RPS16 |
|  |  | 31 | RPS25 |
|  |  | 32 | RPS5 |
|  |  | 33 | SLC25A3 |
|  |  | 34 | SPAG11B |
