# Supplementary figures and images for: Pathogenic landscape of idiopathic male infertility: new insight towards its regulatory networks
Source: NPJ Genom Med. 2016 Aug 17;1:16023–. doi: 10.1038/npjgenmed.2016.23 (PMC5685305; doi:10.1038/npjgenmed.2016.23)

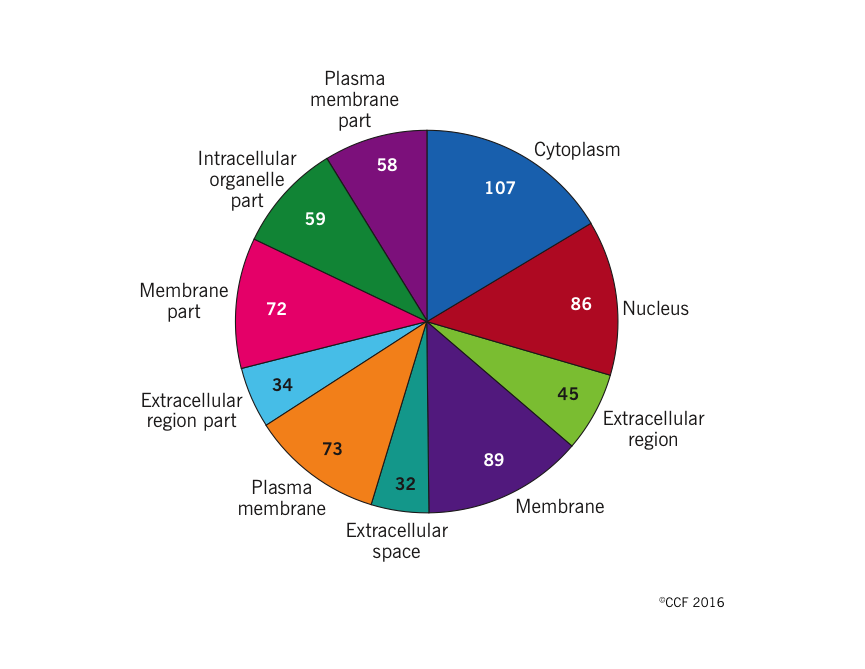

Supplement: Supplementary Figure 1 [file npjgenmed201623-s4.tiff]

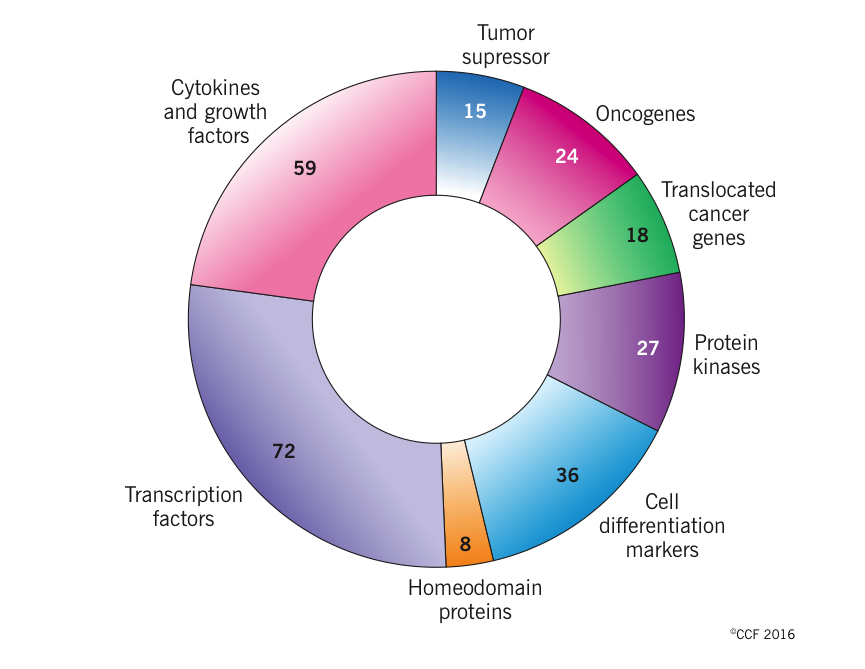

Supplement: Supplementary Figure 2 [file npjgenmed201623-s5.tiff]

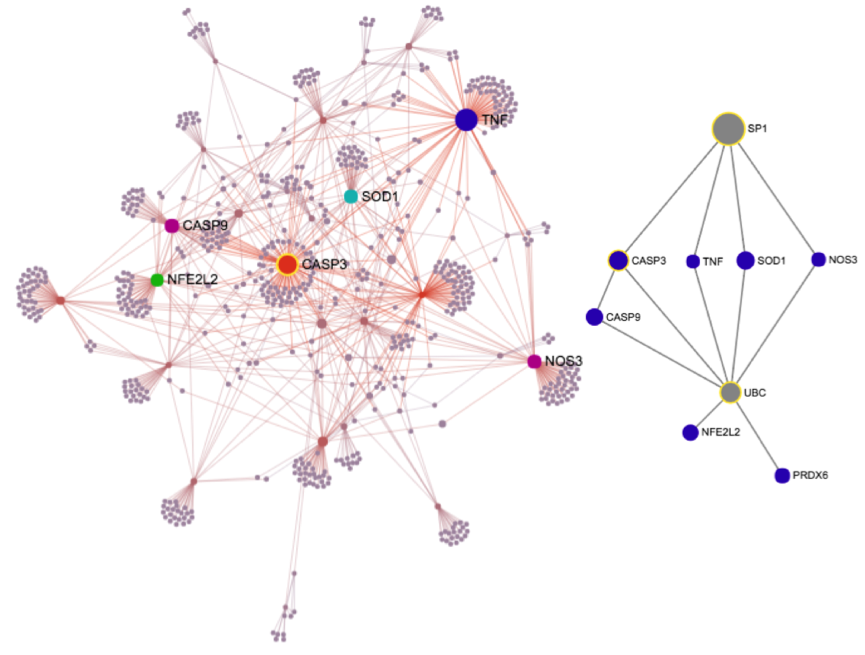

Supplement: Supplementary Figure 3 [file npjgenmed201623-s6.tiff]

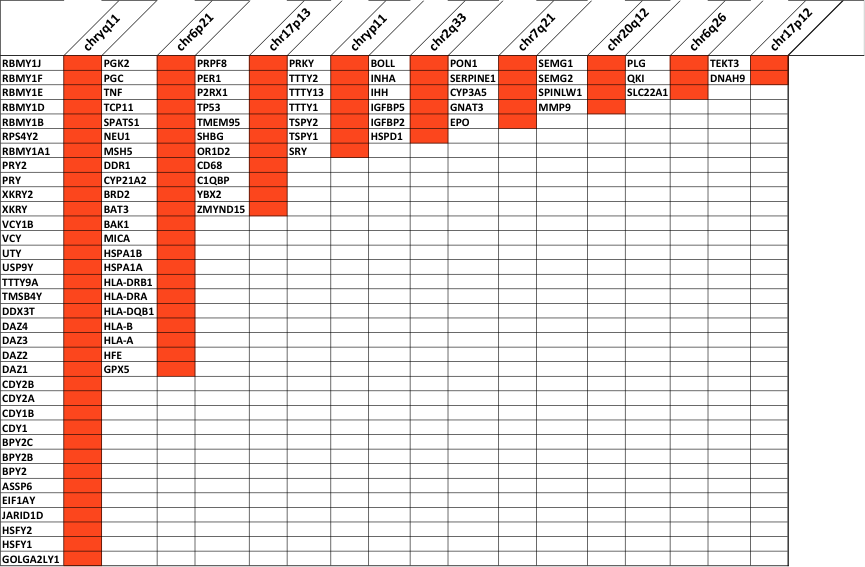

Supplement: Supplementary Figure 4 [file npjgenmed201623-s7.tiff]

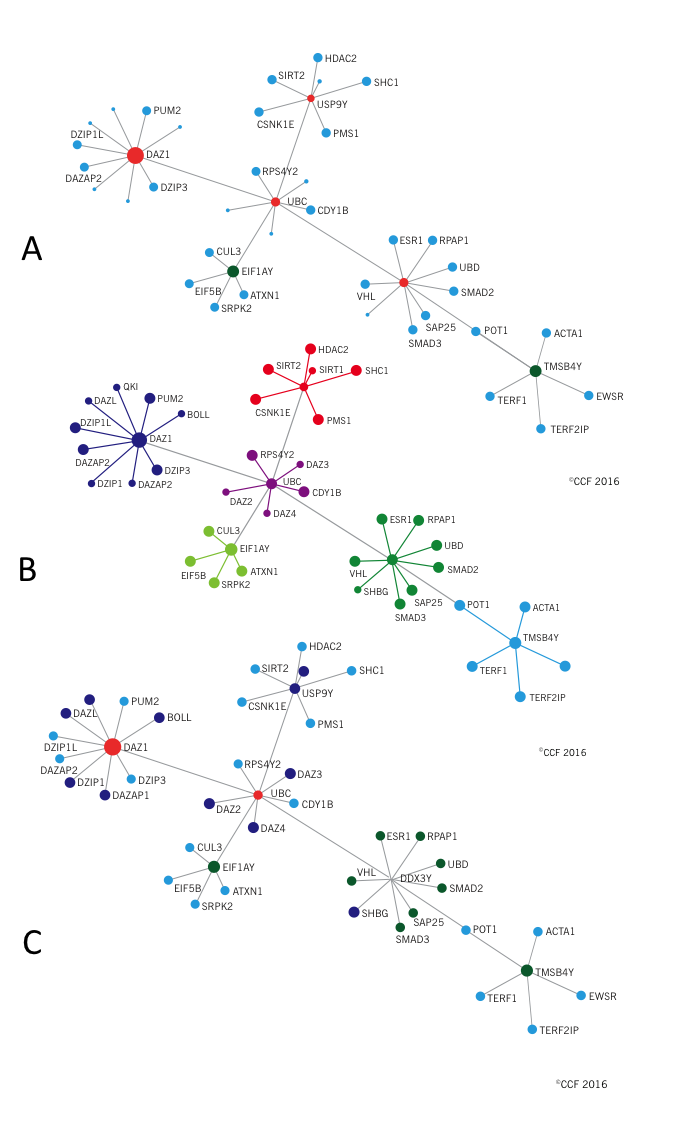

Supplement: Supplementary Figure 5 [file npjgenmed201623-s8.tiff]

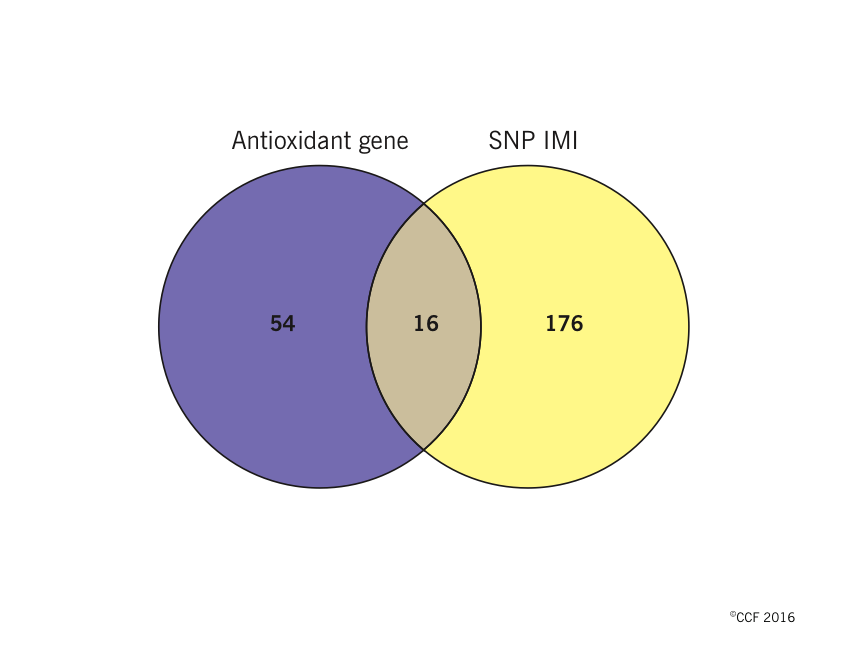

Supplement: Supplementary Figure 6 [file npjgenmed201623-s9.tiff]

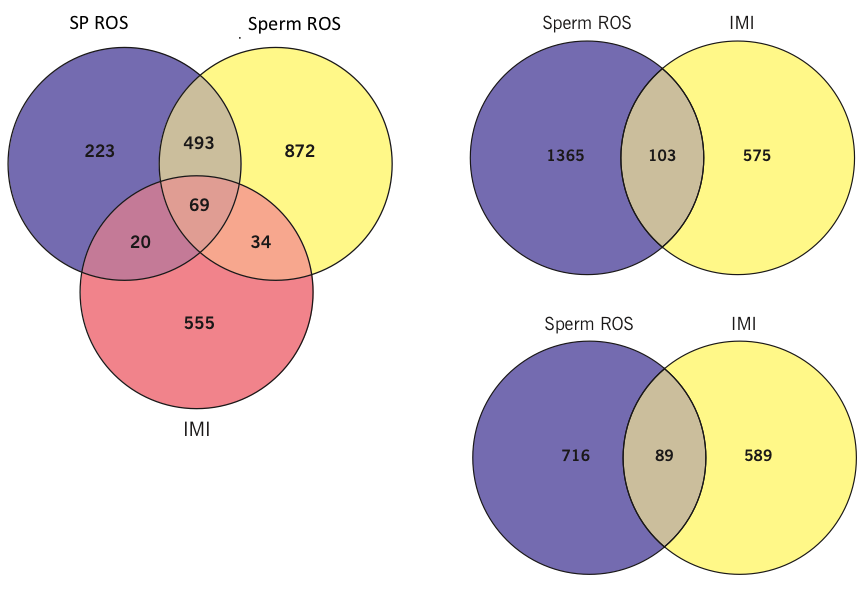

Supplement: Supplementary Figure 7 [file npjgenmed201623-s10.tiff]

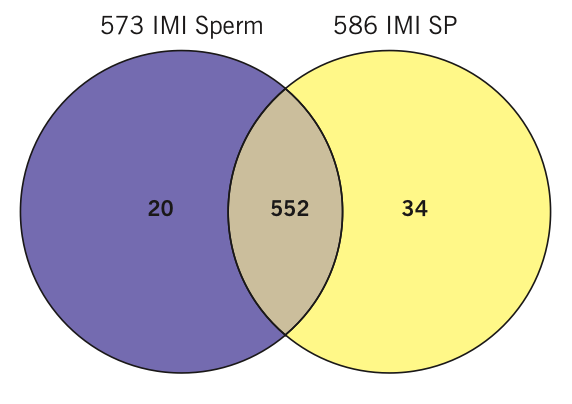

Supplement: Supplementary Figure 8 [file npjgenmed201623-s11.tiff]
